# The Class II/1 anomaly of hereditary etiology vs. Thumb-sucking etiology

**Published:** 2012-06-18

**Authors:** H Pădure, AR Negru, D Stanciu

**Affiliations:** Department of Orthodontics and Dento-facial Orthopedics, "Carol Davila" University of Medicine and Pharmacy, Bucharest

**Keywords:** class II/1, thumb-sucking, hereditary etiology

## Abstract

**Rationale:** The etiology of class II division 1 Angle anomaly comprises many entities, including heredity and the vicious habit of sucking the finger. A close connection between the etiology and the clinical features needs to be outlined, in order to have a more appropriate treatment approach.

**Aim:** This study aims to find common clinical features for two groups of Class II division 1 etiological factors (heredity and the vicious habit of sucking the finger), outlining a characteristic dento-facial pattern for each etiological factor.

**Materials and methods: **160 orthodontic cases were studied, taken randomly from the Department of Orthodontics in "Carol Davila" University of Medicine and Pharmacy, between 2005 and 2011. From a total of 46 diagnosed with Class II/1, the following data were noted for each subject in a table: age, sex, etiology, facial symmetry, profile, size of the lower face, stage lip, labiomentonier ditch, menton, form of arches, palate, occlusion in the three plans, Spee curve, other dental anomalies.

**Discussions:** The facial asymmetry was found in a greater proportion within the finger-sucking group. Subjects in the finger sucking group have 100% pronounced convex profile, with pronounced lip stage and sharp curve of Spee, being known that the thumb-sucking stops the mandible growth and stimulates the upper jaw protrusion.

**Conclusions:** The predominant etiological factor is heredity and the clinical features obvious for II/1 Angle anomaly are present in the highest proportion in the thumb-sucking group, but no significant differences were observed between the groups studied according to etiology.

## Introduction

The etiology of class II division 1 Angle anomaly comprises many entities, including heredity and the vicious habit of sucking the finger [**1-5**]. This study aims to find common clinical features for the two groups of etiological factors, trying to outline a characteristic dento-facial pattern for the II / 1 anomaly of hereditary etiology and one for the thumb-sucking acquired etiology.

## Materials and methods

A total of 160 sheets of orthodontic cases were retrospectively studied, chosen randomly from a pool of patients who showed up at the Department of Orthodontics and Dento-facial Orthopedics of "Carol Davila" University of Medicine and Pharmacy, Bucharest, between 2005 and 2011. 

Among them, 46 patients were considered, specifically those who had the anomaly of Class II division 1 (dental or skeletal); subjects were both male and female, aged between 7 and 23 years old. The following data were noted for each subject in a table: age, sex, etiology, facial symmetry, profile, size of the lower face, stage lip, labiomentonier ditch, menton, form of arches, palate, occlusion in the three plans, Spee curve, other dental anomalies. 

All the data needed was taken from photographs, panoramic radiographs, lateral cephalograms, casts and medical sheets. All the lateral cephalograms were analyzed by the Tweed-Merriefield method.

## Results 

**Fig. 1:**
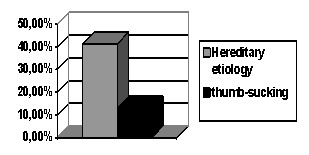
Proportions of the two etiological factors studied

Out of the total subjects studied, 46 had the anomaly of class II / 1 Angle, which represents a rate of 28.75%; the subjects were aged between 7 and 23 years old, of whom, 63% were girls and the rest boys. The hereditary etiology was found in 41.3% of the cases, while the thumb-sucking etiology was present in 13% (**Fig. 1**).

**Fig. 2:**
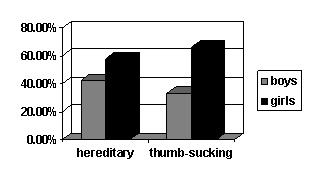
Sex distribution

The distribution according to sex in the two etiology groups indicates a relative balance in terms of heredity (42.1% boys and 57.9% girls) and a more significant difference regarding the thumb sucking: 66.7%; 33.3% boys and girls respectively (**Fig. 2**). 

**Fig. 3:**
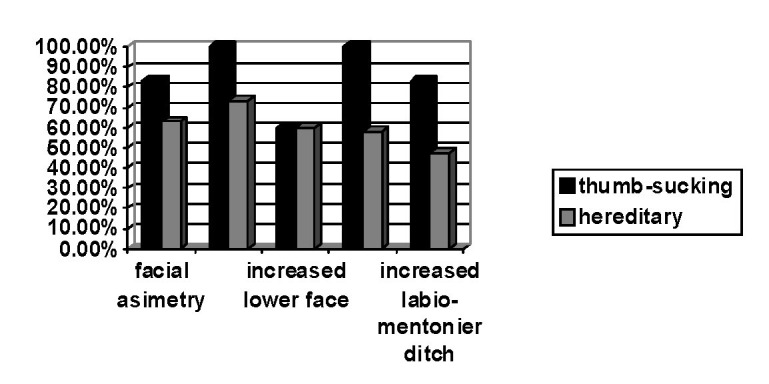
Facial features

The facial exam revealed a pronounced convex profile for all the patients in the thumb-sucking group with pronounced lip stage and a sharp curve of Spee (**Fig. 3**).

**Fig. 4:**
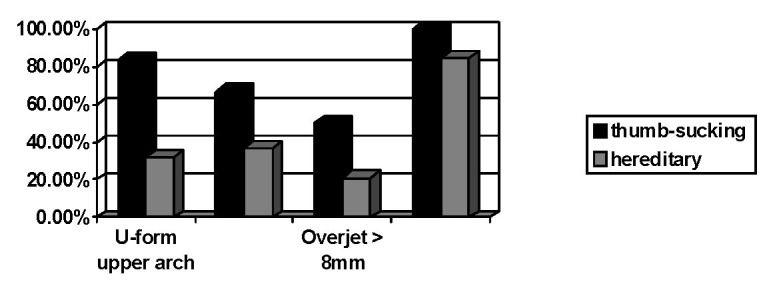
The most pregnant thumb-sucking group

Regarding the arches’ form for the hereditary etiology group, the U-shaped upper arch was present in 31.59% of the cases, but the V-shaped arches (21.05%), 15.8% pentagon, parable (10.52%), were also noted; the thumb-sucking group had the upper arch “U” shaped at a rate of 83,33%, the rest being V-shaped (**Fig. 4**). 

As expected [**5,6**], very deep palate was observed in 66.67% of the cases of those who sucked the finger, compared to only 36.84% within the hereditary etiology group. Regarding occlusion, the overjet marks a considerable difference statistically, registering over 50% of the cases with more than 8 mm overjet among those who have sucked the finger and only 21% in the hereditary etiology group. An increased curve of Spee was noted for 84,21% of the hereditary etiology group and 100% among those who have sucked the finger (**Fig. 4**).

## Discussions

Regarding gender distribution, there are some differences within the thumb sucking group, more girls being registered - 66, 7%, the frequency being perhaps due to the emotional factor present in females [**8,9**].

The facial asymmetry in a greater proportion within the group that is finger sucking is a predictable result [**7-14**], given the nature of this vicious habit, not done symmetrically in most of the times [**10-12**].

It was noted that the subjects in the finger-sucking group have a 100% pronounced convex profile, with a pronounced lip stage and sharp curve of Spee, the results being expected, taking into account that thumb-sucking stops the mandible growth [**12**] and stimulates the upper jaw protrusion [**13**] and vestibular inclination, or even the position of the upper incisors [**15**]. This group is also distinguished by the presence of a greater overjet, frequently more than 8 mm.

There was an equal percentage in terms of increased lower face, which was observed in both groups of subjects – a very unusual result, given the typical profile of this anomaly [**15-17**], most often with a decreased lower face. Our result may have turned out this way, taking into account the young age - many subjects were below 12 years old - and being known that the mandible angle closes until the age of 12 [**17**].

## Conclusions

Both heredity and thumb-sucking are etiological factors occurring in patients who have the same features of II / 1 Angle anomaly, the frequency of this anomaly being present in almost one third of all cases in this study, which are consistent with international literature data [**15-17**]. The predominant etiological factor is the hereditary one [**2,4**] and the clinical features obvious for II / 1 Angle anomaly are present in the highest proportion within the thumb-sucking group, but no significant differences were observed between the groups studied according to the etiology.
